# Supplementary material for: Conductive and elastic bottlebrush elastomers for ultrasoft electronics
Source: Nat Commun. 2023 Feb 4;14:623. doi: 10.1038/s41467-023-36214-8 (PMC9899285; doi:10.1038/s41467-023-36214-8)
Supplement: Supplementary file 1 — Supplementary_Information [file 41467_2023_36214_MOESM1_ESM.pdf]

# Supplementary Information for

## Conductive and elastic bottlebrush elastomers for ultrasoft electronics

Pengfei Xu, Shaojia Wang, Angela Lin, Hyun-Kee Min, Zhanfeng Zhou, Wenkun Dou, Yu Sun, Xi Huang, Helen Tran*, Xinyu Liu*

*Corresponding authors. Email: tran@utoronto.ca; xyliu@mie.utoronto.ca

**This PDF file includes:**

Supplementary Figs. 1 to 35
Supplementary Tables 1 to 4
Supplementary References

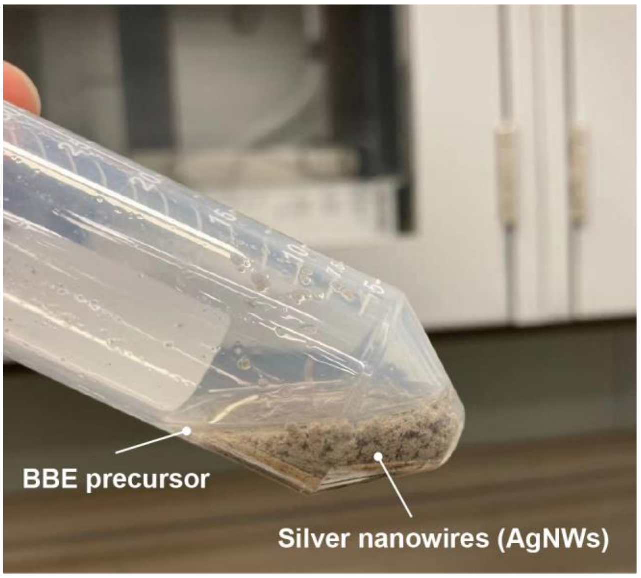


**Supplementary Fig. 1.** Use of metal-based fillers can be limited by the difficulty of material preparations. For instance, silver nanowires (AgNWs) cannot be dispersed into the BBE precursor, due to the polarity difference of AgNWs and BBE. The surface ligand of AgNWs is polyvinylpyrrolidone (PVP), which is polar and hydrophilic, while the BBE is hydrophobic. Thus, the AgNWs settled down and formed clusters in the BBE precursor. Such a poor dispersity failed to make BBE conductive.

**Supplementary Table 1. Comparison of different methods of preparing bottlebrush elastomers.**

| Bottlebrush elastomers | Method | Side chains | Crosslinker | Commercially available | Young's modulus | Reference |
|---|---|---|---|---|---|---|
| PDMS BBE | Free radical polymerization | PDMS (MCR-M11) | PDMS (DMS-R18, DMS-R22) | All materials are commercially available | 0.84 - 65.48 kPa | [1–4] |
| PDMS BBE | Hydrosilylation + platinum catalyst | PDMS (MCR-H21) | PDMS (DMS-H25) | All materials are commercially available | 7.43 - 132 kPa | [5,6] |
| | | PDMS (MCR-V21, MCR-V25) | PDMS-based crosslinker | | 1.2 - 7.4 kPa | [7] |
| Linear-bottlebrush-linear elastomers | Atom transfer radical polymerization (ATRP) | PDMS, PMMA | Aggregation of linear polymer tails | No | 2.1 - 155.7 kPa | [1,8–10] |
| PDMS-r-PEG elastomers | ATRP | PDMS, PEG | Isocyanate:hydroxyl (NCO:OH) and isocyanate:amine (NCO:NH2) coupling | No | 1.8 - 27.8 kPa | [11] |
| ROMP PDMS BBE | Ring-opening metathesis polymerization (ROMP) | PDMS | Bis-benzophenone–based PDMS photocrosslinker | No | 6.2 - 92 kPa | [12] |
| Poly(4-methylcaprolactone) (P4MCL) BBE | Ring-opening metathesis polymerization (ROMP) | P4MCL | 4,4′-bioxepane-7,7′-dione | No | 10 - 100 kPa | [13,14] |
| PDMS-stat-PEO BBE | Ring-opening metathesis polymerization (ROMP) | PDMS, PEO | Bis-benzophenone–based PDMS photocrosslinker | No | 32 kPa and 7.7 kPa | [15] |

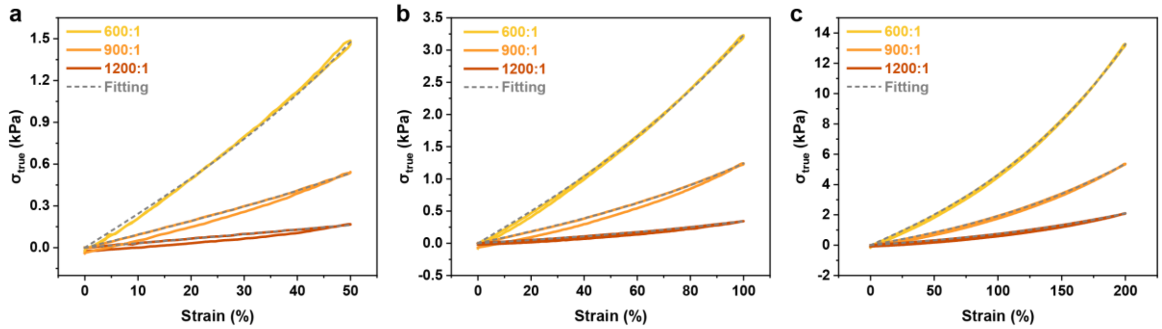


**Supplementary Fig. 2.** Cycling tests of PDMS BBEs with different crosslinking ratios (MM:CL = 600:1, 900:1, and 1200:1) at the strain of **a** 50%, **b** 100%, and **c** 200%, respectively. The dash line represents the fitting curve obtained from the model for unentangled polymer networks. The fitting model is:

$$\sigma_{true} = \frac{E}{9}[(\varepsilon+1)^2 - (\varepsilon+1)^{-1}]\left\{1 + 2\left[1 - \frac{\beta[(\varepsilon+1)^2 - (\varepsilon+1)^{-1}]}{3}\right]\right\}$$

where $\beta$ is the strand-extension ratio and $E$ is the structural Young's modulus, and $\sigma_{true}$ and ε are true stress and strain, respectively.

**Supplementary Table 2. Fitting parameters of PDMS BBEs with different crosslinking ratios at the strain of 400%.**

| **Molar ratio of monomer:crosslinker** | ***E* (kPa)** | ***β*** | ***ε*** |
|---|---|---|---|
| 600:1 | 1.787±0.182 | 0.034±0.003 | 400% |
| 900:1 | 1.078±0.158 | 0.029±0.003 | 400% |
| 1200:1 | 0.789±0.198 | 0.030±0.002 | 400% |

The fitting parameters (*E* and *β*) might differ with the selected strain. From the cycling tensile tests, the elongation-at-break of PDMS BBEs should be higher than 400% while 400% strain is the highest strain we selected to conduct the cycling tensile tests. Thus, this table shows the fitting parameters at the strain of 400%.

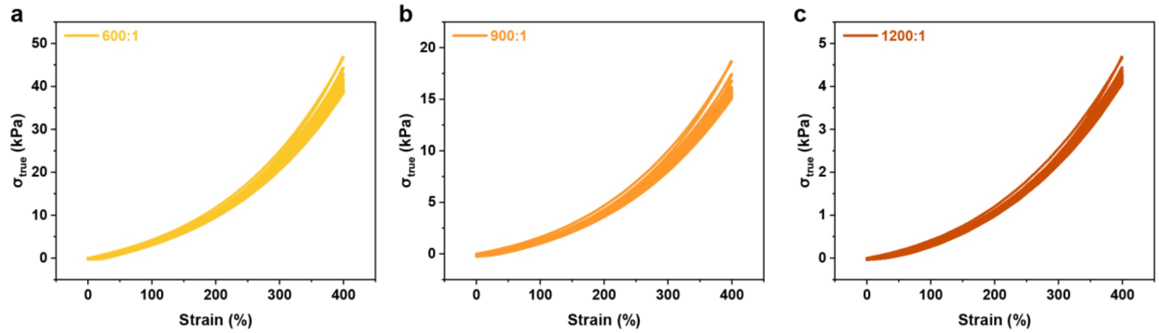


**Supplementary Fig. 3.** Loading-unloading tests of PDMS BBEs with different crosslinking ratios (MM:CL = **a** 600:1, **b** 900:1, and **c** 1200:1) for 20 cycles under the strain of 0 – 400%. The strain rate is 0.070 $s^{-1}$.

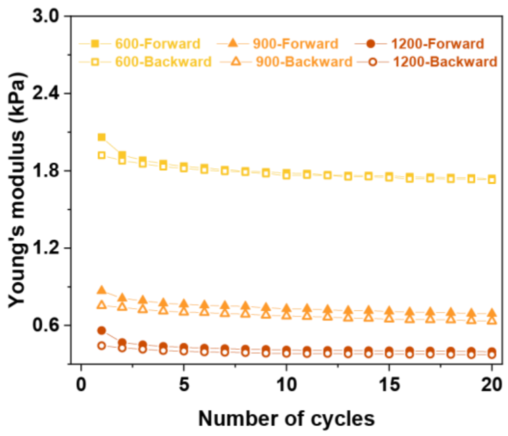


**Supplementary Fig. 4.** The change of Young's modulus for each loading-unloading cycle of three representative PDMS BBE samples with different crosslinking ratios (MM:CL = 600:1, 900:1, and 1200:1). "Forward" represents the Young's modulus of forward stretching, "Backward" represents the Young's modulus of backward releasing. The strain rate is 0.070 $s^{-1}$.

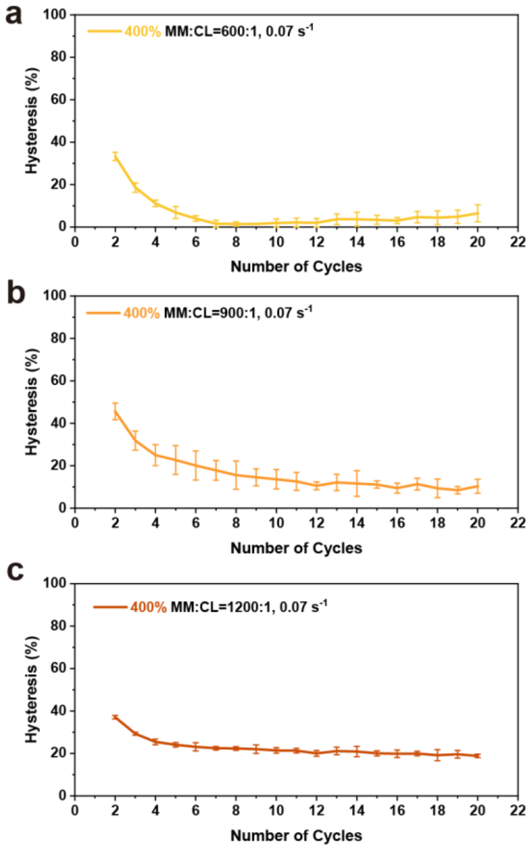


**Supplementary Fig. 5.** The change of hysteresis for each loading-unloading cycle of PDMS BBEs with different crosslinking ratios (**a** MM:CL = 600:1, **b** 900:1, and **c** 1200:1) under the strain of 0 – 400%. The strain rate is 0.070 $s^{-1}$. Error bars denote the standard deviation of the measurements.

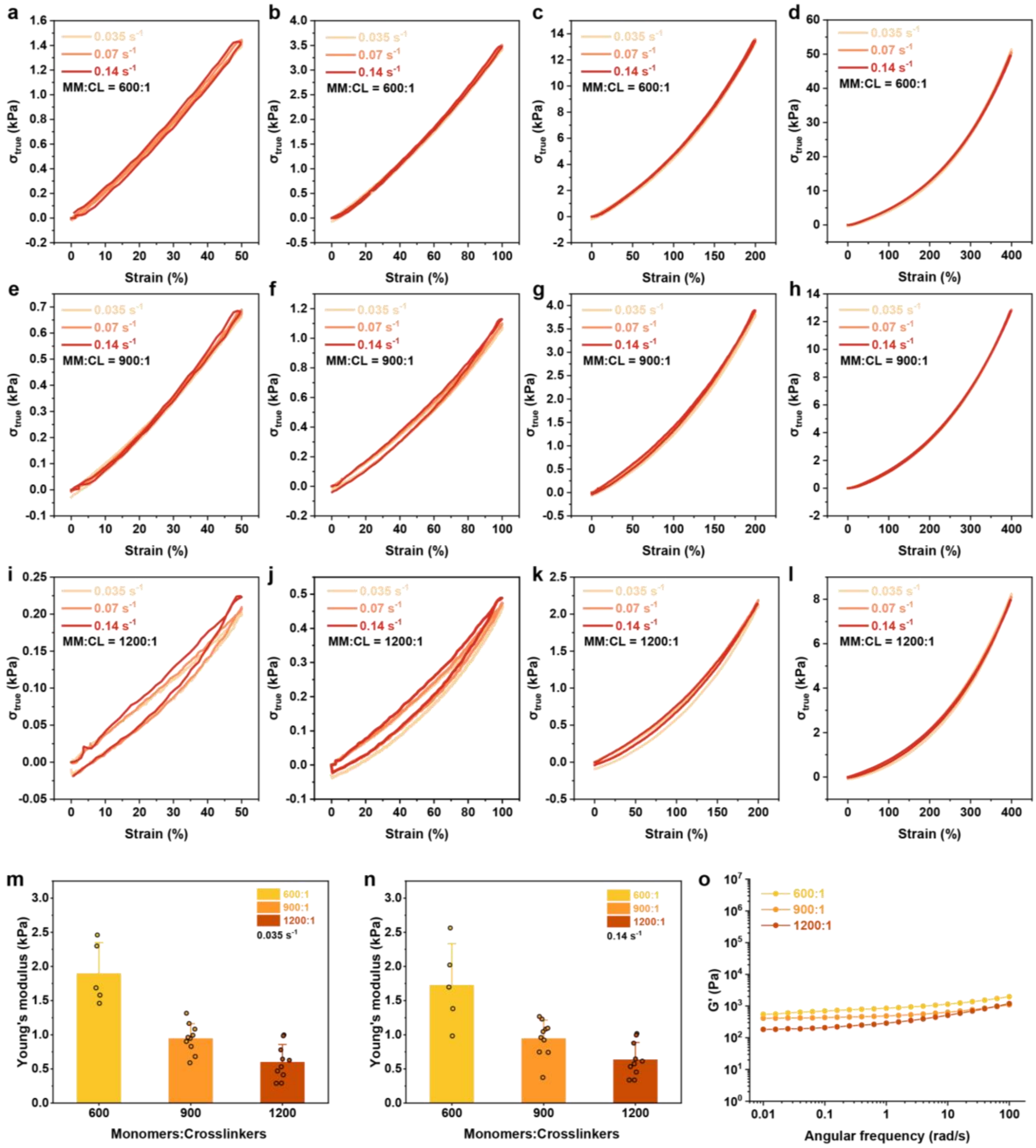


**Supplementary Fig. 6. a-d** Cycling tests of PDMS BBEs (MM:CL = 600:1) under strain rates of 0.035 $s^{-1}$, 0.070 $s^{-1}$, and 0.14 $s^{-1}$. **e-h** Cycling tests of PDMS BBEs (MM:CL = 900:1) under the strain rate of 0.035 $s^{-1}$, 0.070 $s^{-1}$, and 0.14 $s^{-1}$. **i-l** Cycling tests of PDMS BBEs (MM:CL = 1200:1) under the strain rate of 0.035 $s^{-1}$, 0.070 $s^{-1}$, and 0.14 $s^{-1}$. At different strain rates, the elastomers were stretched to 0 - 50%, 0 -

100%, 0 - 200%, and 0 - 400%, respectively. **m-n** The calculated Young's modulus of BBEs under strain rates of 0.035 $s^{-1}$ and 0.14 $s^{-1}$. **o** Storage modulus as a function of sweeping frequency of pure BBEs with different crosslinking ratios. The dynamic frequency sweeps show a nearly linear elastic regime from 0.01 rad/s to 1 rad/s. At lower crosslinking levels (e.g., 1200:1), there is a non-negligible modulus increase at higher frequency (>1 rad/s), possibly due to the low crosslinking level that induces relaxations of polymer. However, all the strain rates (25 mm/min: 0.035 $s^{-1}$, 50 mm/min: 0.070 $s^{-1}$, 100 mm/min: 0.14 $s^{-1}$) used in this work are within the elastic regime (0.01 to 1 rad/s: 0.0016 to 0.16 $s^{-1}$). Error bars denote the standard deviation of the measurements.

**Supplementary Table 3. The summary of Young's modulus and elastic range of different elastomers.**

Electrospun TPUe fiber mats
SBR:BR
Cu-CPU
SBR:BR/CNT
SBR:BR/CNT/EtOH
PU fiber
PU/PEDOT:PSS
polyurethane
PU/PS1(PS2)

Ecoflex
Silbione® RTV 4528 1:1
CH=CH2 - SH PDMS
HO - Si PDMS
HO - H PDMS
CH=CH2 - H PDMS
PDMS–MPUx–IU1−x
Silbione® RTV 4420 (stiffer) 1:1
Sylgard 184 (15:1)
Sylgard 186 (10:1)
SMI G/G
Sylgard 184 (10:1)
Sylgard 184 (5:1)
PDMS-TFB

Natural elastomer
SBR
IIR
VHB4910
liquid crystal elastomers
ABA/ABC elastomers

PDMS-stat-PEO bottlebrush elastomer
BBE (MM:CL = 600:1)
BBE (MM:CL = 900:1)
BBE (MM:CL = 1200:1)

| Young's modulus (MPa) | Elastic range | Elastomers | | Reference |
|---|---|---|---|---|
| **PU-based elastomers** | | | | |
| ~0.6 | 200% | Electrospun TPUe fiber mats | | 16 |
| ~2 | 100% | SBR:BR (styrene butadiene rubber:butadiene rubber) | unfilled | 17 |
| ~3.2 | 50% | | 3 phr CNT + ethanol | |
| ~2.4 | 75% | | 3 phr CNT | |
| >2 | 100% | Cu–dimethylglyoxime–urethane-polyurethane (CPU) elastomer | | 18 |
| ~10 | 200% | PU/PEDOT:PSS | PU fiber | 19 |
| ~20 | 50% | | PU/PEDOT:PSS | |
| 45 | 300% | polyurethane | | 20 |
| 390 | 200% | polyurethane (PU) network crosslinked by crown-ether-based pseudo[2]rotaxane (PS1) and PS2 | | 21 |
| **Silicone-based elastomers** | | | | |
| >0.055 | 200% | Ecoflex | | 22 |
| 0.845 | 90% | Silicone-based elastomers | Sylgard 186 (10:1) | 23 |
| 1.5 | 90% | | SMI G/G | |

| Young's modulus (MPa) | Elastic range | Elastomers | | Reference |
|---|---|---|---|---|
| 0.048 | 90% | | Silbione® RTV 4528 1:1 | |
| 0.455 | 90% | | Silbione® RTV 4420 (stiffer) 1:1 | |
| 0.06 | 100% | PDMS elastomers | CH=CH2 - SH PDMS | [24] |
| 0.13 | 100% | | HO - Si PDMS | |
| 0.18 | 100% | | HO - H PDMS | |
| 0.4 | 100% | | CH=CH2 - H PDMS | |
| 0.43 - 0.71 | 50% | PDMS–MPUx–IU1−x | | [25] |
| 2.081 | 41% | Sylgard 184 | Sylgard 184 (5:1) | [26] |
| 1.955 | 42.9% | | Sylgard 184 PDMS (10:1) | |
| 0.75 | 51.5% | | Sylgard 184 PDMS (15:1) | |
| 56.4 | 40% | PDMS-TFB | | [27] |
| **Others** | | | | |
| 3.3 - 5.9 | 300% | Natural elastomer | poly(cis-1,4-isoprene) elastomer | [28] |
| 2.1 - 10.3 | <25% | SBR elastomers | poly(styrene-co-butadiene) | [29] |
| 3 - 3.4 | 200% | Butyl rubber (IIR) | | |
| ~0.2 | 300% | VHB4910 | | [30] |
| ~0.5 | ~50% | Polydomain nematic main-chain liquid crystal elastomers | | [31] |
| 0.7 | 300% | atactic polypropylene, poly(ethylene-co-propylene), and poly-(ethylene-co-1-hexene) ABA/ABC elastomers | | [32] |
| **Bottlebrush elastomers** | | | | |

| Young's modulus (MPa) | Elastic range | Elastomers | Reference |
|---|---|---|---|
| 0.032 | 250% | PDMS-stat-PEO bottlebrush elastomer | [33] |
| 0.00186 | 400% | BBE (MM:CL = 600:1) | This work |
| 9.60E-04 | 400% | BBE (MM:CL = 900:1) | |
| 6.30E-04 | 400% | BBE (MM:CL = 1200:1) | |

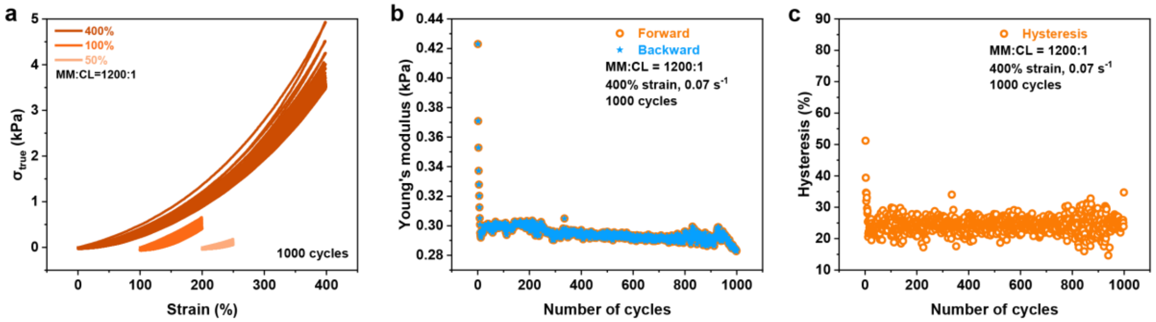


**Supplementary Fig. 7. a** Long term cycling tests (1000 cycles at the strain of 400%, 100%, and 200%, respectively) of PDMS BBEs with the crosslinking ratio of MM:CL = 1200:1. **b** The change of Young's modulus for each loading-unloading cycle of PDMS BBEs (MM:CL = 1200:1) under the strain of 0 – 400%. **c** The change of hysteresis for each loading-unloading cycle of PDMS BBEs (MM:CL = 1200:1) under the strain of 0 – 400%. 1000 cycles were conducted with the strain rate of 0.070 $s^{-1}$. "Forward" represents the Young's modulus of forward stretching, "Backward" represents the Young's modulus of backward releasing. The strain rate is 0.070 $s^{-1}$.

a

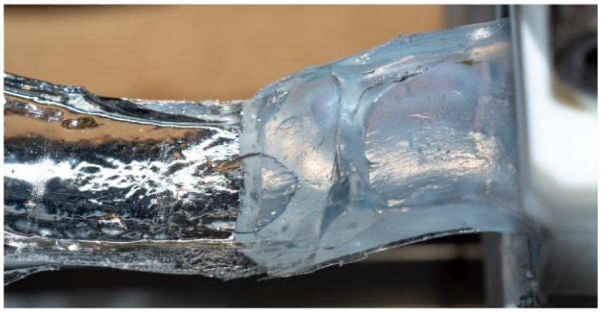

b

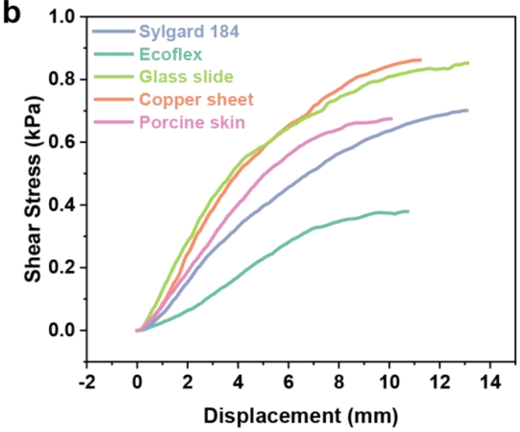


**Supplementary Fig. 8. a** Photographs of the adhesion test between PDMS BBEs (MM:CL = 1200:1) and Ecoflex. **b** Curves for shear strength versus displacement of PDMS BBEs (MM:CL = 1200:1) adhered on different substrates.

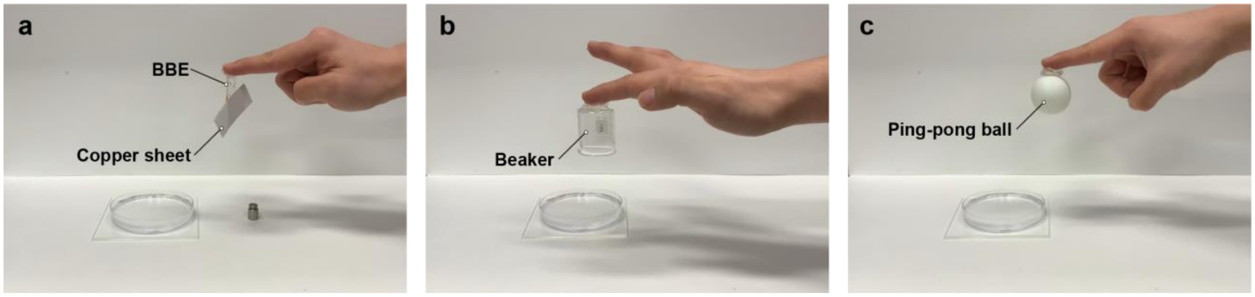


**Supplementary Fig. 9. a-c** Photographs of adhering PDMS BBEs (MM:CL = 1200:1) between the skin and copper sheet, beaker, and ping-pong ball, showcasing the adhesive property of PDMS BBEs.

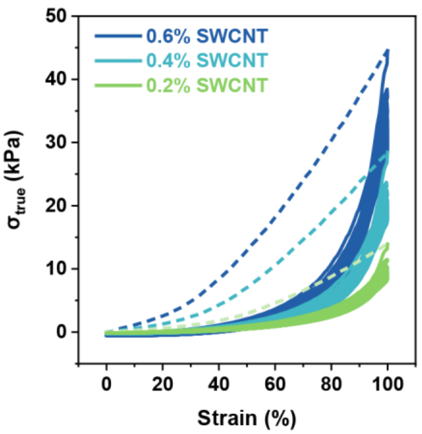


**Supplementary Fig. 10.** Cycling tests of PDMS BBEs with 0.2 wt%, 0.4 wt%, and 0.6 wt% SWCNT at the strain of 100%. The strain rate is 0.070 $s^{-1}$. 20 cycles were conducted, with the first half cycle represented by the dash line.

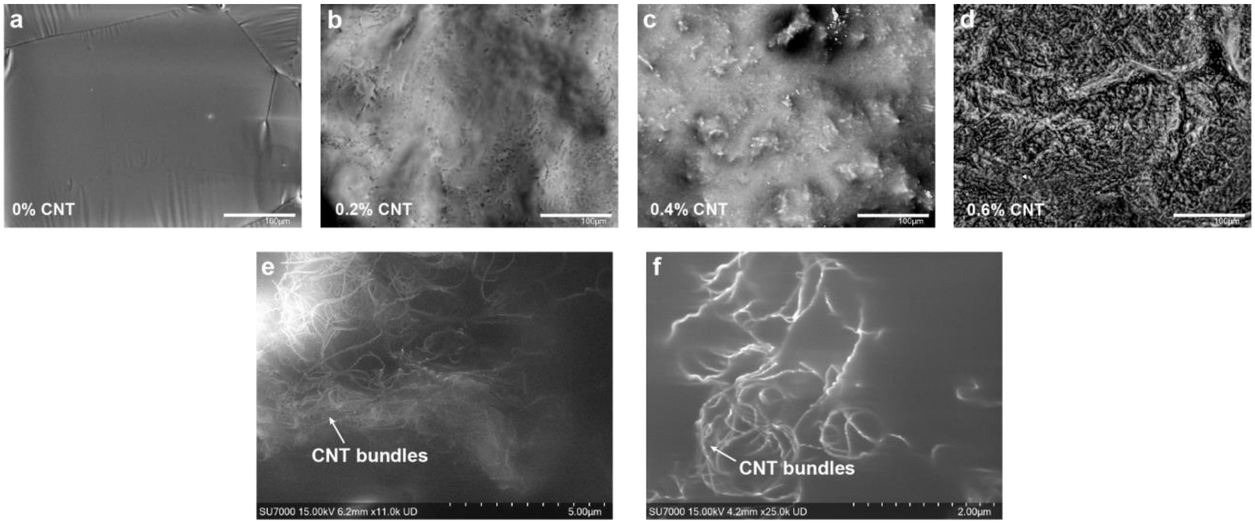


**Supplementary Fig. 11.** SEM images of the surfaces of **a** pure BBEs and BBEs with **b** 0.2 wt%, **c** 0.4 wt%, and **d** 0.6 wt%. The scale bar is 100 μm. SEM images at **e** 11000× and **f** 25000× of the cross-sectional surfaces of 0.4 wt% SWCNT/BBE samples.

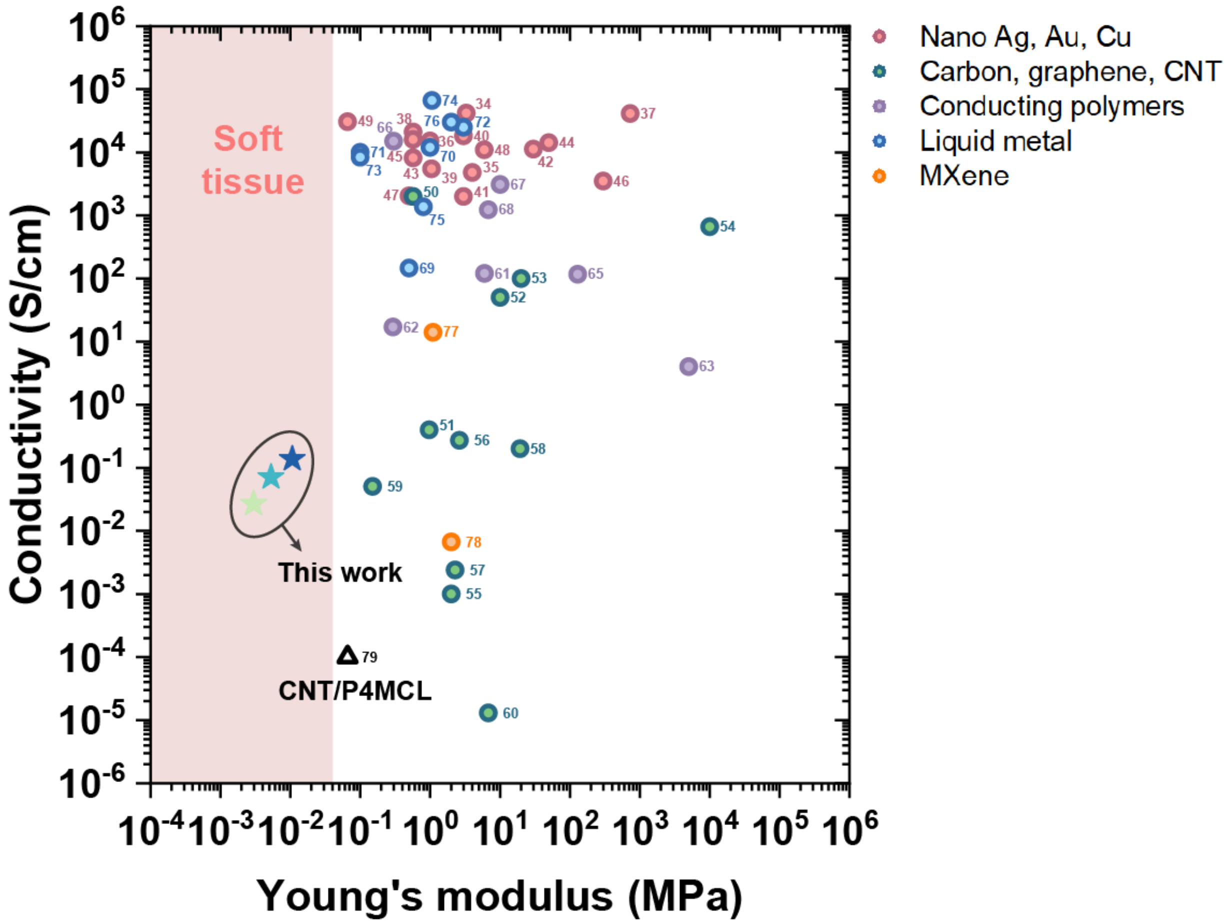


**Supplementary Fig. 12.** The Ashby-style plot of conductivity and Young's modulus of different conductive elastomers with metallic nanomaterials, carbon-based materials, conducting polymers, liquid metal, and MXene as conductive components. Hydrogels are not included as they contain water and are not solvent-free. The SWCNT/BBE in this work shows the lowest Young's modulus among all other elastomers.

**Supplementary Table 4. The summary of Young's modulus and conductivity of conductive materials based on different matrix materials and conductive fillers.**

| Young's modulus (MPa) | Conductivity (S/cm) | Matrix materials | Conductive materials | Reference |
|---|---|---|---|---|
| **Nano Ag, Au, Cu** | | | | |
| 3.28 | 41850-72600 | SBS-elastomer | Ag-Au nanowires | [34] |
| ~4 | 4800 | TPU nanofiber | AgNWs | [35] |
| >1 | 15100 | PDMS | Ag flakes | [36] |
| 731.5 | 41245 | PU | Ag nanoflowers | [37] |
| 0.57-3.7 | 21000 | PDMS+PS membrane | AgNPs | [38] |
| 1.05 | 5450 | SBS-elastomer | AgNPs | [39] |
| >3 | 18168 | PDMS | AgNPs | [40] |
| ~1 | 2000 | poly(styrene-block-isoprene-block-styrene) (SIS) | AgNPs | [41] |
| 0.00339-30 | 11210 | SBS-elastomer | ligand-exchange AgNWs | [42] |
| 0.57-3.7 | 8130 | PDMS | AgNWs | [43] |
| 49.7 | 14205 | PU | AgNWs | [44] |
| 0.57-3.7 | 16000 | PDMS and PVA layer | Au-TiO2 nanowires | [45] |
| >300 | 3500 | LBL or VAF | AuNPs | [46] |
| >0.5 | 2040 | Dragon skin or Ecoflex | Cu-An nanowire | [47] |
| 5.94 | 10990 | Polyurethane Fibers | Ag nanoflowers | [48] |

| Young's modulus (MPa) | Conductivity (S/cm) | Matrix materials | Conductive materials | Reference |
|---|---|---|---|---|
| 0.06585 | 30485 | PU fiber | AgNPs formed in-situ | 49 |
| **Carbon, graphene, CNT** | | | | |
| 0.57-3.7 | 2000 | PDMS | SWCNT | 50 |
| 0.97-6.55 | 0.4 | S-SBR | carbon black+silica microparticles | 51 |
| ~10 | 50 | Perhexane-25B | long SWCNT | 52 |
| ~20 | 100 | fluorinated copolymer rubber Daiel-G912 | long SWCNT | 53 |
| >10000 | 660 | fiber | Graphene oxide | 54 |
| 2 | 0.001 | natural rubber | MWCNT | 55 |
| 2.62 | 0.271 | TU-PDMS | CNT | 56 |
| 2.28 | 0.0024 | SBR | MWCNT | 57 |
| 19.4 | 0.2 | PU | CNT forests | 58 |
| 0.15-0.4 | 0.000017-0.0505 | S-LSR | MWCNT | 59 |
| 6.8 | 0.000013 | NR-RGs | CNT | 60 |
| **Conducting polymers** | | | | |
| >6 | 120 | PU | PEDOT:PSS | 61 |
| 0.293 | 17 | P123: Poly(ethylene glycol)−block−poly(propylene glycol)−block−poly(ethylene glycol) | PEDOT:PSS | 62 |
| 5010 | 4 | TFB | P3BT | 63 |

| **Young's modulus (MPa)** | **Conductivity (S/cm)** | **Matrix materials** | **Conductive materials** | **Reference** |
|---|---|---|---|---|
| 0.57-3.7 | 550 | PDMS | PEDOT:PSS | [64] |
| 128.9 | 116.3 | Polycaprolactone- block - polytetrahydrofuran- block - polycaprolactone (PCTC) | polypyrrole | [65] |
| 0.3-0.6 | 15000 | SEBS elastomer | PANI | [66] |
| >10 | 3100 | PEDOT:PSS | PEDOT:PSS + ionic liquid | [67] |
| 6.8 | 1230 | PEDOT:PSS | PEDOT:PSS+PEO+ Zonyl | [68] |
| **Liquid metal** | | | | |
| ~0.5 | 146.05 | Commercially available textile | Liquid metal | [69] |
| ~1 | 12000 | SBS | Liquid metal | [70] |
| [NO_PRINTED_FORM].1 | 10000 | spinning SBS | liquid metal | [71] |
| ~3 | 25000 | PEVA | hydrogen-doped liquid metal | [72] |
| 0.1 | 8331 | EVA | liquid metal + Ag flakes | [73] |
| 1.06 | 66500 | PVA | AgNP laryer + EGaIn layer | [74] |
| 0.8 | 1370 | PDMS | liquid metal | [75] |
| 2-300,000 | 30000 | PDMS, Ecoflex, glass | liquid metal particles | [76] |
| **MXene** | | | | |
| 1.1 | 14 | Natural rubber | Mxene | [77] |
| 2-14 | 0.00667 | nitrile butadiene rubber(NBR) | modified Mxene | [78] |
| **CNT** | | | | |

| **Young's modulus (MPa)** | **Conductivity (S/cm)** | **Matrix materials** | **Conductive materials** | **Reference** |
|---|---|---|---|---|
| 0.066 | 1.00E-04 | BBE | CNT/P4MCL | [79] |
| 0.00298 | 0.0268 | BBE | 0.2 wt% SWCNT | This work |
| 0.00529 | 0.0708 | BBE | 0.4 wt% SWCNT | |
| 0.01065 | 0.1378 | BBE | 0.6 wt% SWCNT | |

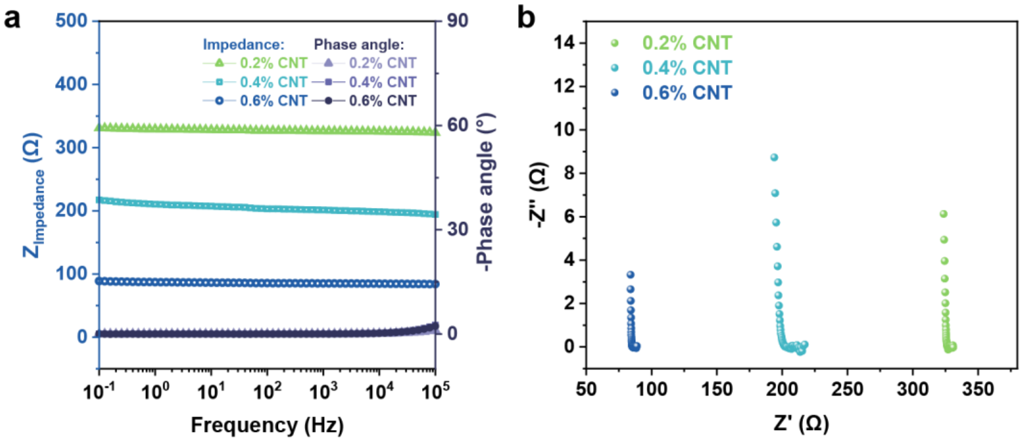


**Supplementary Fig. 13. a** Bode plot and **b** Nyquist plot of impedance measurements of PDMS BBEs with different concentrations of SWCNT (0.2 wt%, 0.4 wt%, and 0.6 wt%). The impedance decreases with the increase of CNT concentration.

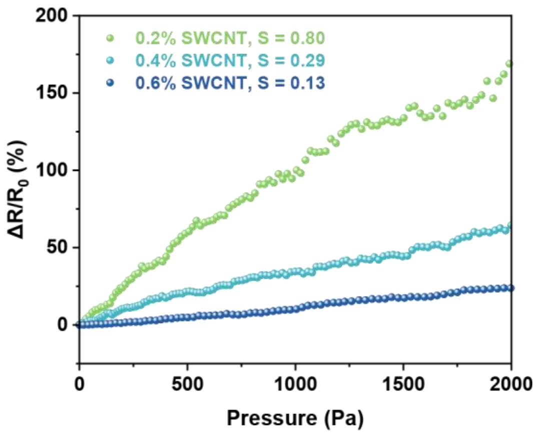


**Supplementary Fig. 14.** Normalized change in resistance as a function of compressing pressure. The sensitivity of SWCNT/BBE with 0.2 wt%, 0.4 wt%, and 0.6 wt% CNT is 0.80, 0.29, and 0.13, respectively.

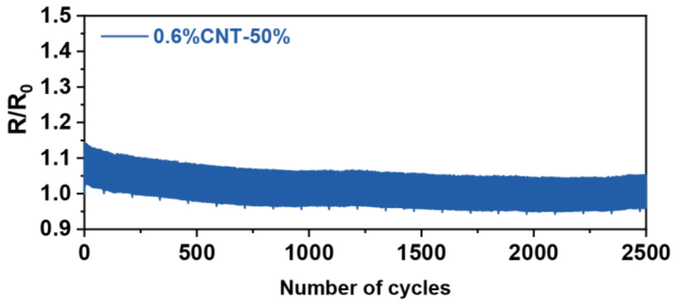

**Supplementary Fig. 15.** Cyclic durability (2500 cycles) of the normalized change in resistance for BBE with 0.6 wt% CNT under cyclic loading to 100% strain.

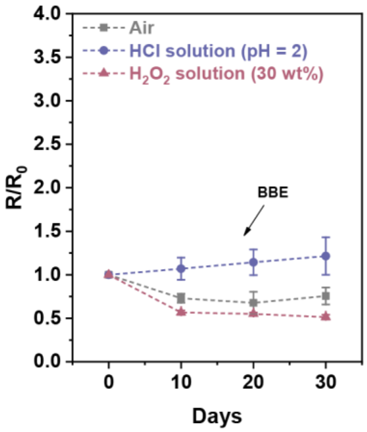


**Supplementary Fig. 16.** A zoom-in figure of Fig. 3g, showcasing the limited change in resistance being found in SWCNT/BBE after stored in HCl solution, $H_2O_2$ solution, or exposed to air for 30 days. Error bars denote the standard deviation of the measurements.

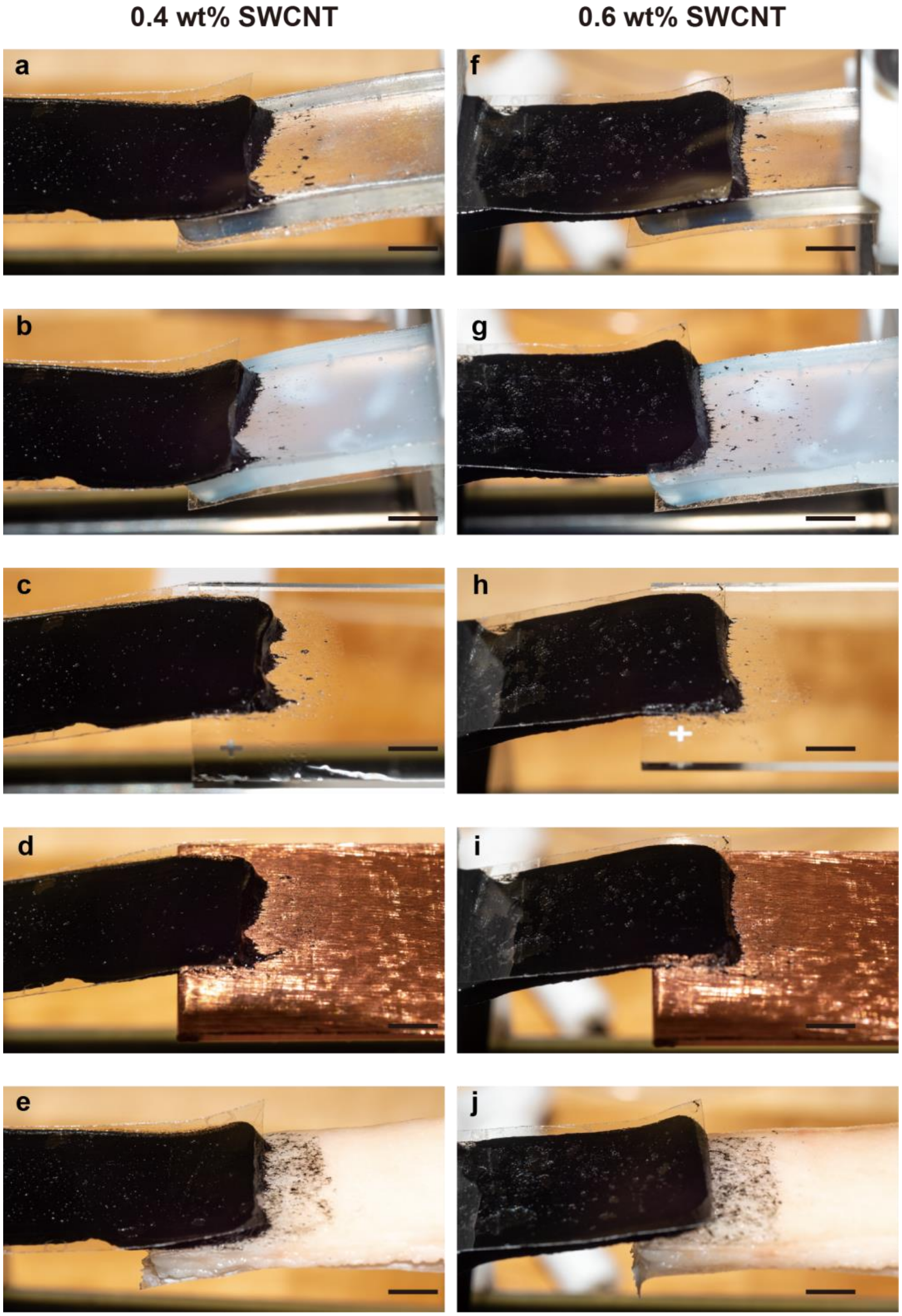


**Supplementary Fig. 17.** Photographs of the adhesion test between SWCNT/BBE (**a-e** 0.4 wt% and **f-j** 0.6 wt% CNT) and different substrates (Sylgard 184, Ecoflex 00-10, glass slide, copper sheet, and porcine skin). The scale bar is 5 mm.

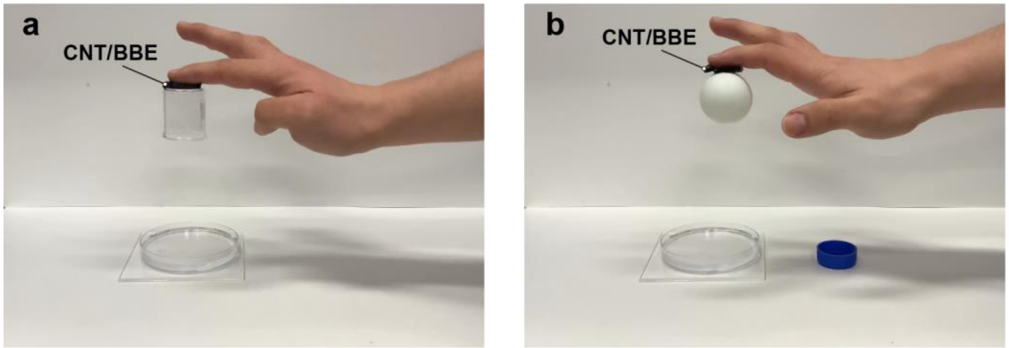

**Supplementary Fig. 18. a-b** Photographs of adhering SWCNT/BBE (0.4 wt% CNT) between the skin and a beaker, or a ping-pong ball, showcasing the adhesive property of SWCNT/BBE.

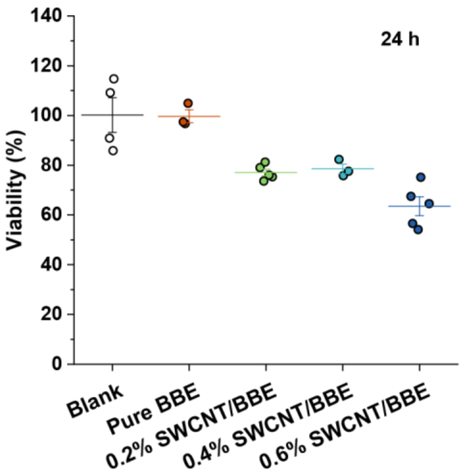


**Supplementary Fig. 19.** Cell viability of human dermal fibroblast cells exposed to pure BBE and conductive BBE (0.2 wt%, 0.4 wt%, and 0.6 wt% SWCNT) relative to counts in the blank control group. Error bars denote the standard deviation of the measurements.

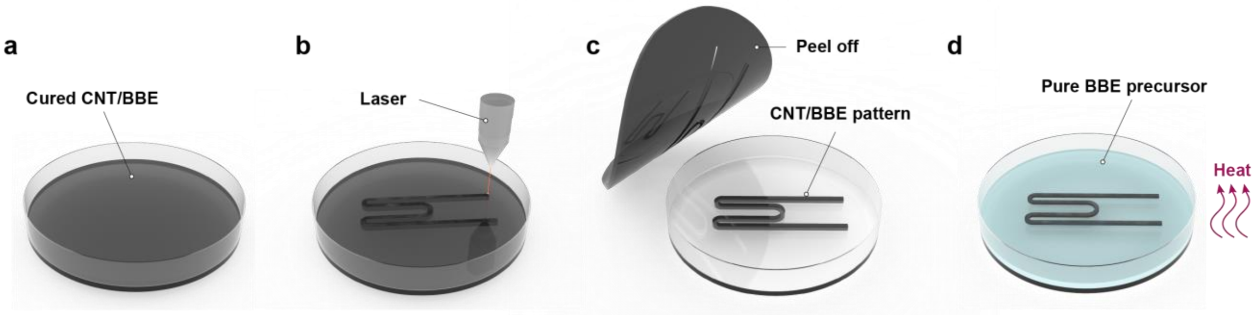


**Supplementary Fig. 20. Laser cutting method to pattern SWCNT/BBE and make electronics with insulators. a** First, the SWCNT/BBE were cured in a petri dish. **b** Next, the SWCNT/BBE pattern was formed by laser cutting. **c** Then, the cut area was left in the petri dish by peeling off surrounded materials. **d** Finally, the SWCNT/BBE pattern was covered by pure BBE precursor to form the insulator layer after curing under heat.

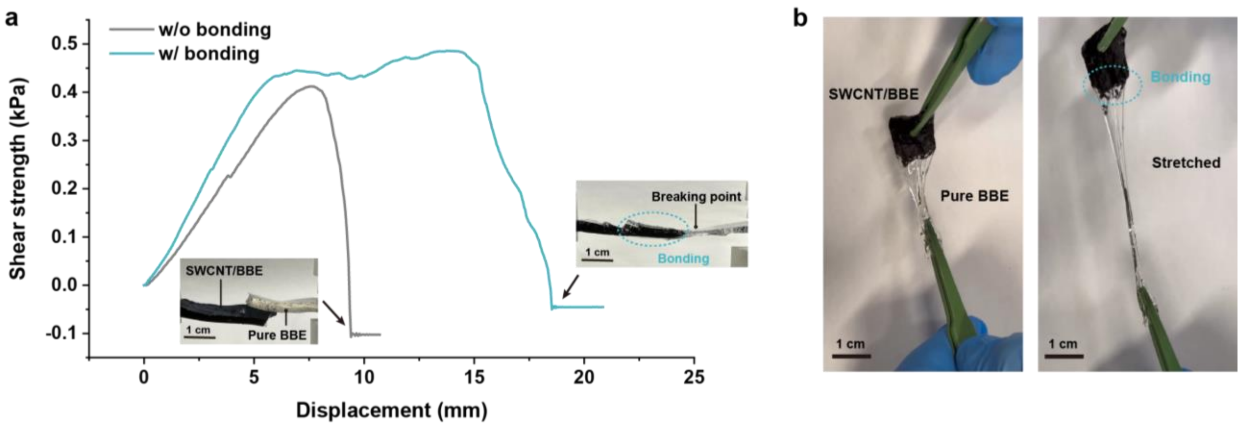


**Supplementary Fig. 21. a** The shear strength versus stretching displacement measured between the SWCNT/BBE and pure BBE. The bonding was formed by sequentially curing the SWCNT/BBE and pure BBE, while physically attaching two pieces of samples cannot form bonding at the interface. The inset photographs show the lap-shear test of the two cases. **b** Photographs of the bonded SWCNT/BBE and pure BBE. No detaching was found upon stretching.

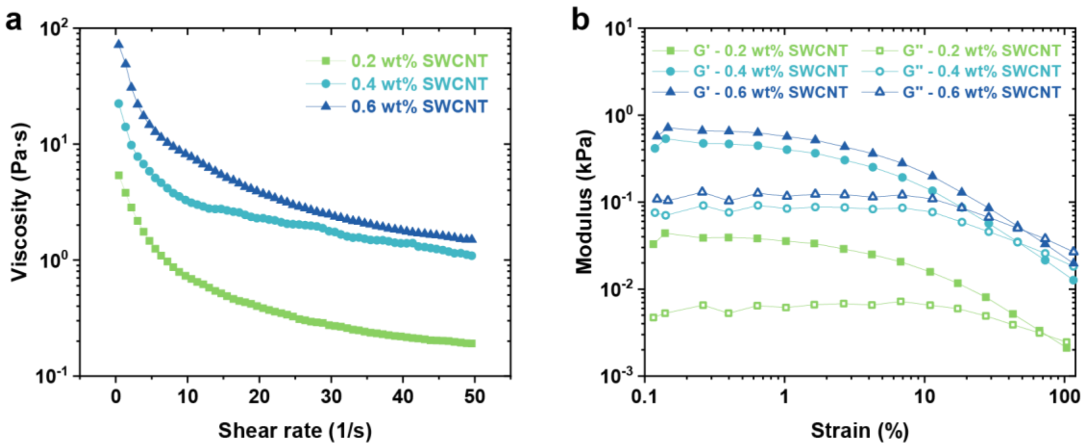


**Supplementary Fig. 22. a** Frequency dependent viscosity and **b** amplitude sweep of BBE containing different concentrations of CNT.

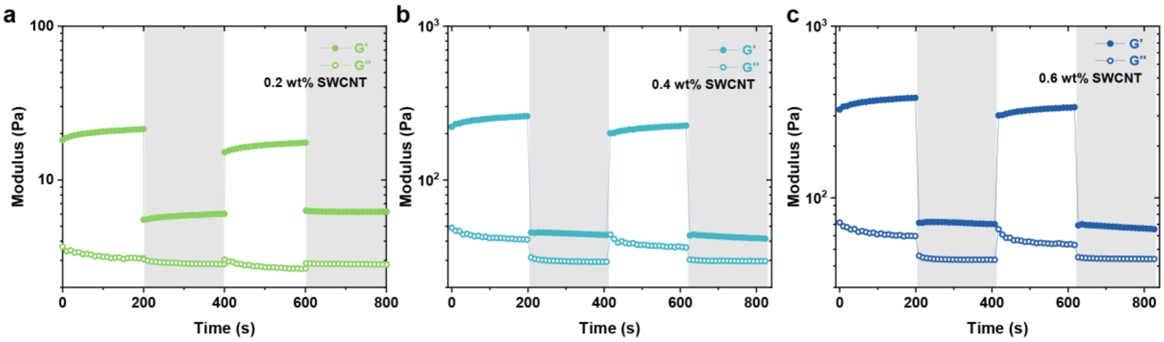


**Supplementary Fig. 23.** Storage modulus and loss modulus of BBE with **a** 0.2 wt% CNT, **b** 0.4 wt% CNT, and **c** 0.6 wt% CNT under alternatively changed strain of 1% and 50%.

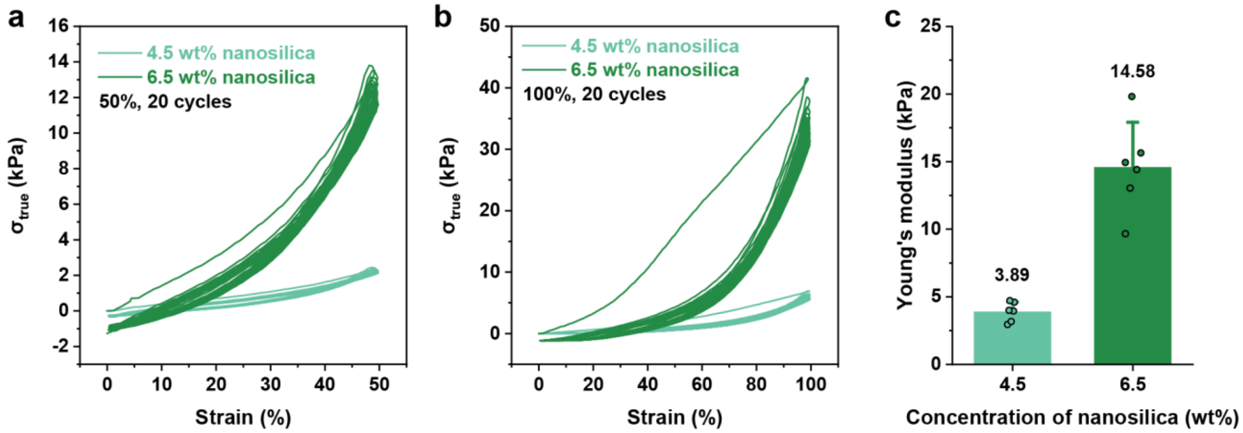


**Supplementary Fig. 24.** Mechanical characterizations of nanosilica/BBE materials. **a** Cycling tests of PDMS BBEs with 4.5 wt% and 6.5 wt% nanosilica at the strain of 50% and **b** 100%. 20 cycles were conducted at the strain rate of 0.070 $s^{-1}$. **c** The Young's modulus BBE with 4.5 wt% nanosilica (average of 3.89 kPa), and 6.5 wt% nanosilica (average of 14.58 kPa). The Young's modulus was calculated by linear fitting of 0 – 10% strain. Error bars denote the standard deviation of the measurements.

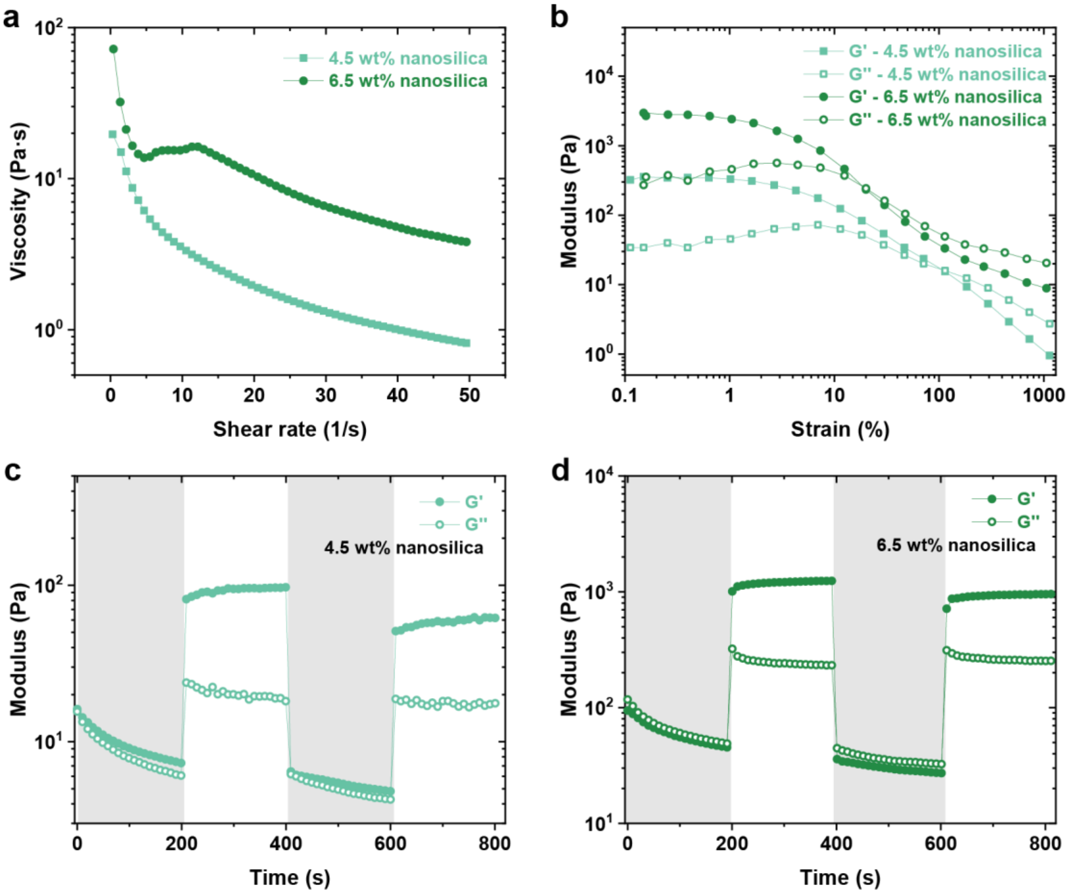


**Supplementary Fig. 25. Rheological properties of fumed silica/BBE inks. a** Frequency dependent viscosity and **b** amplitude sweep of BBE containing different concentrations (4.5 wt%, 6.5 wt%) of nanosilica. **c** Storage modulus and loss modulus of BBE with 4.5 wt% CNT and **d** 0.6 wt% nanosilica under alternatively changed strain of 50% and 1%.

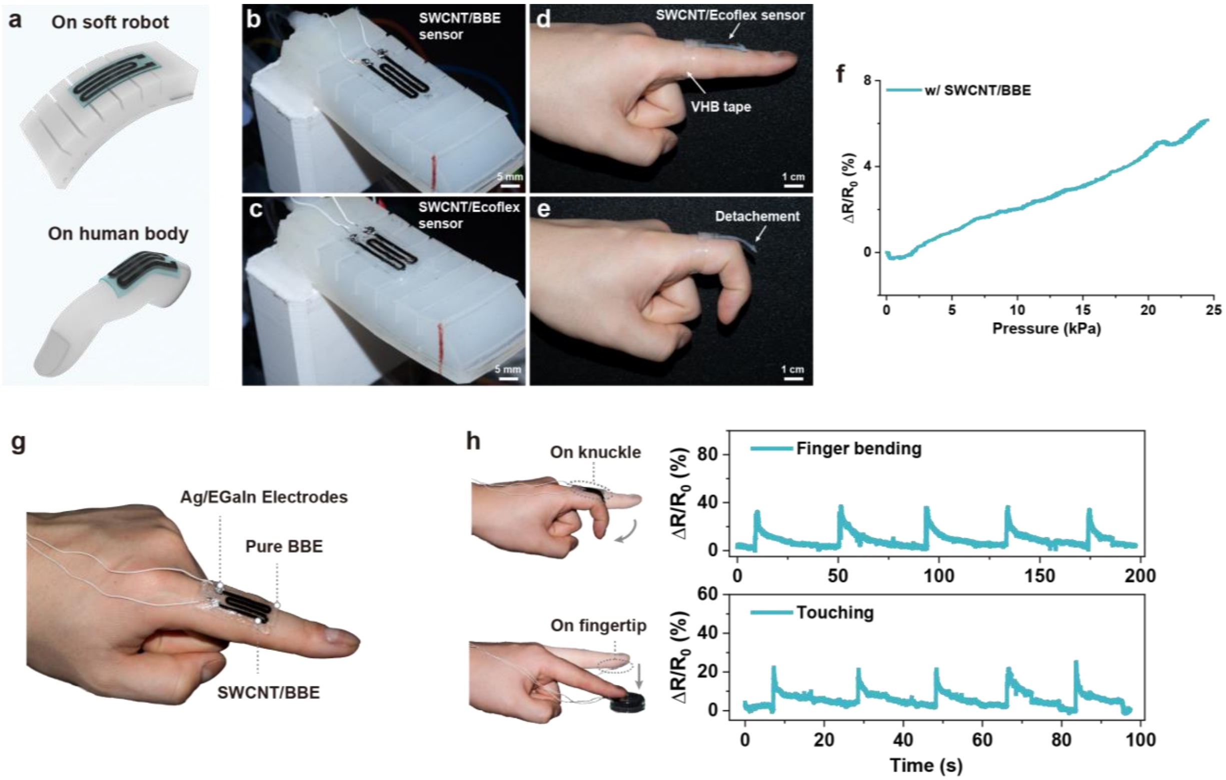


**Supplementary Fig. 26. a** A schematic of the laser-cut strain sensor attached on soft robot and human body due to its self-adhesive property. **b** Laser-cut SWCNT/BBE sensor and **c** SWCNT/Ecoflex sensor on the Ecoflex-based actuator. The SWCNT/BBE sensor was attached on the actuator based on self-adhesive property, while the SWCNT/Ecoflex sensor was attached on the actuator by the epoxy resin. **d-e** When bending the finger, the SWCNT/Ecoflex sensor detached with the skin due to poor adhesion of Ecoflex. **f** Normalized change in resistance of the SWCNT/BBE sensor as a function of inflated pressure applied on the pneumatic actuator **g** Photograph of the laser-cut strain sensor adhered on the knuckle of a human hand. **h** The response of the strain sensor on the knuckle or fingertip, when bending the finger or touching a petri dish with the fingertip, respectively.

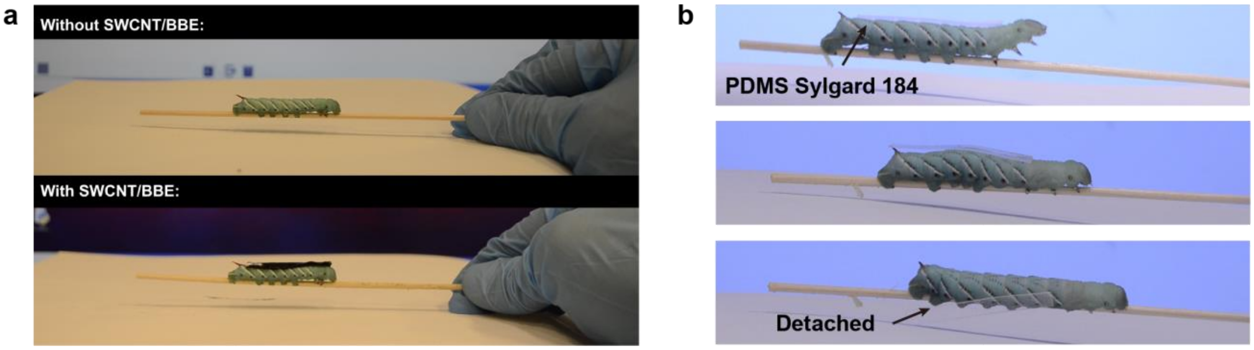


**Supplementary Fig. 27. a** Photographs of the hornworm without attached with the SWCNT/BBE sensor and the one attached with the SWCNT/BBE sensor. **b** Photographs of the hornworm attached with the PDMS Sylgard 184. The PDMS Sylgard 184 can be easily detached from the hornworm during its crawling due to high stiffness and low adhesion.

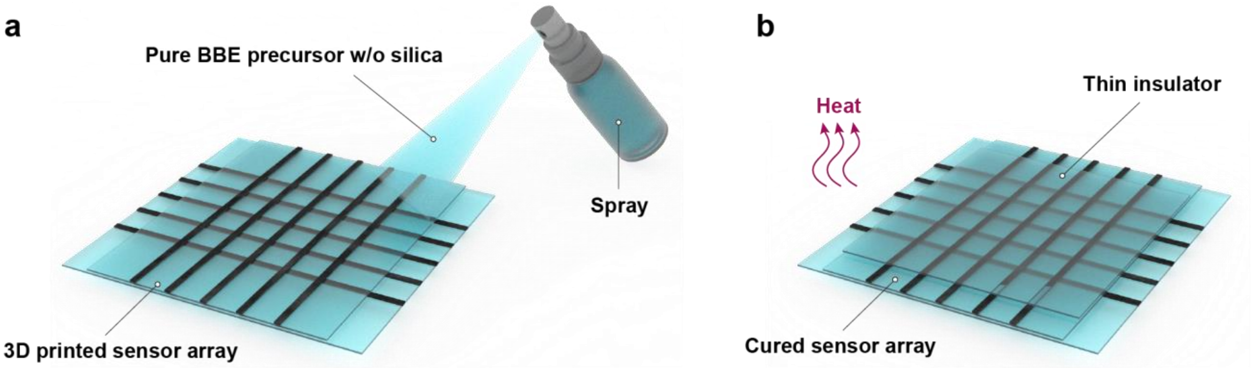


**Supplementary Fig. 28. Schematic showing spraying coating process of the insulator.** As an alternative method of preparing the top insulator besides 3D printing, the BBE w/o additives (i.e., silica) can be spray coated. **a** The pure BBE precursors were loaded into the spray, and coated on the 3D printed sensor array. **b** After the BBE insulator was coated on the sensor array, the full device was then cured under heat.

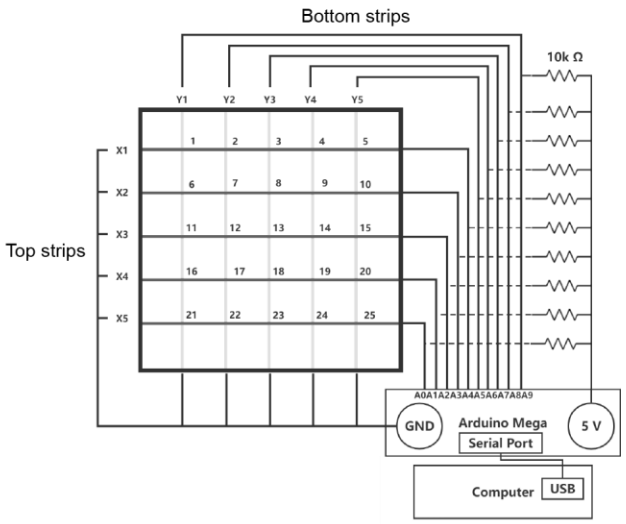


**Supplementary Fig. 29. Schematic of the circuit for the sensor array.** x1, x2, x3, x4, x5 represent the 5 top sensor strips. y1, y2, y3, y4, y5 represented the 5 bottom sensor strips. The crossed 10 sensor strips generated 25 sensing positions through connecting with 10 resistors (1 kΩ), an Arduino and a computer for data collection.

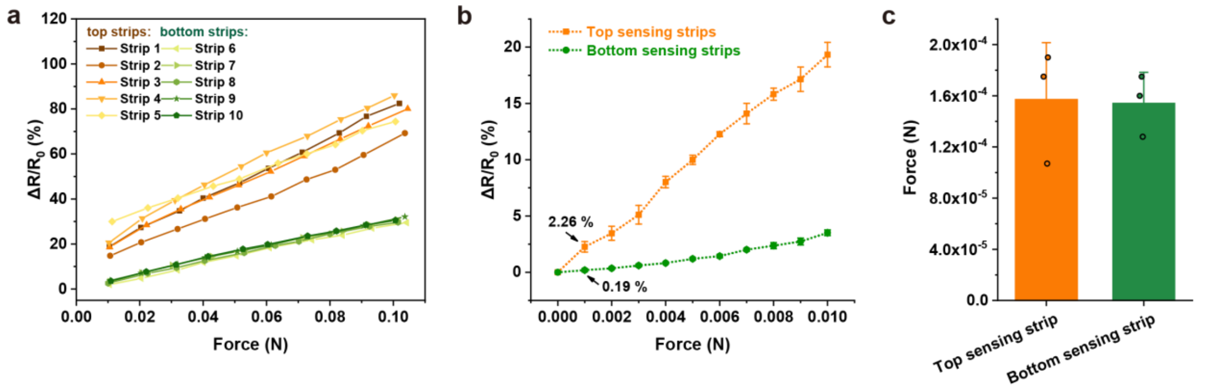


**Supplementary Fig. 30.** **a** Calibration of ten sensor strips of the sensing strips in the range of 0.01-0.1 N, with normalized resistance change of sensor strips at the top and bottom, respectively, as a function of applied force. **b** The average of normalized resistance change of typical sensing strips in the calibration range of 0-0.01 N. **c** Measured forces for the blank groups (when the normalized resistance change is 0). The mean forces and standard deviations are: 0.000157 ± 0.000044 N (top sensing strip), and 0.000154 ± 0.000024 N. Based on the three sigma limits statistical calculation, the limit of detection for the top sensing strip is: 0.000157 + 3×0.000044 = 0.00029 N, and the limit of detection for the bottom sensing strip is: 0.000154 + 3×0.000024 = 0.00023 N. Thus, the limit of detection for the touch pad is determined to be 0.00029 N. Error bars denote the standard deviation of the measurements.

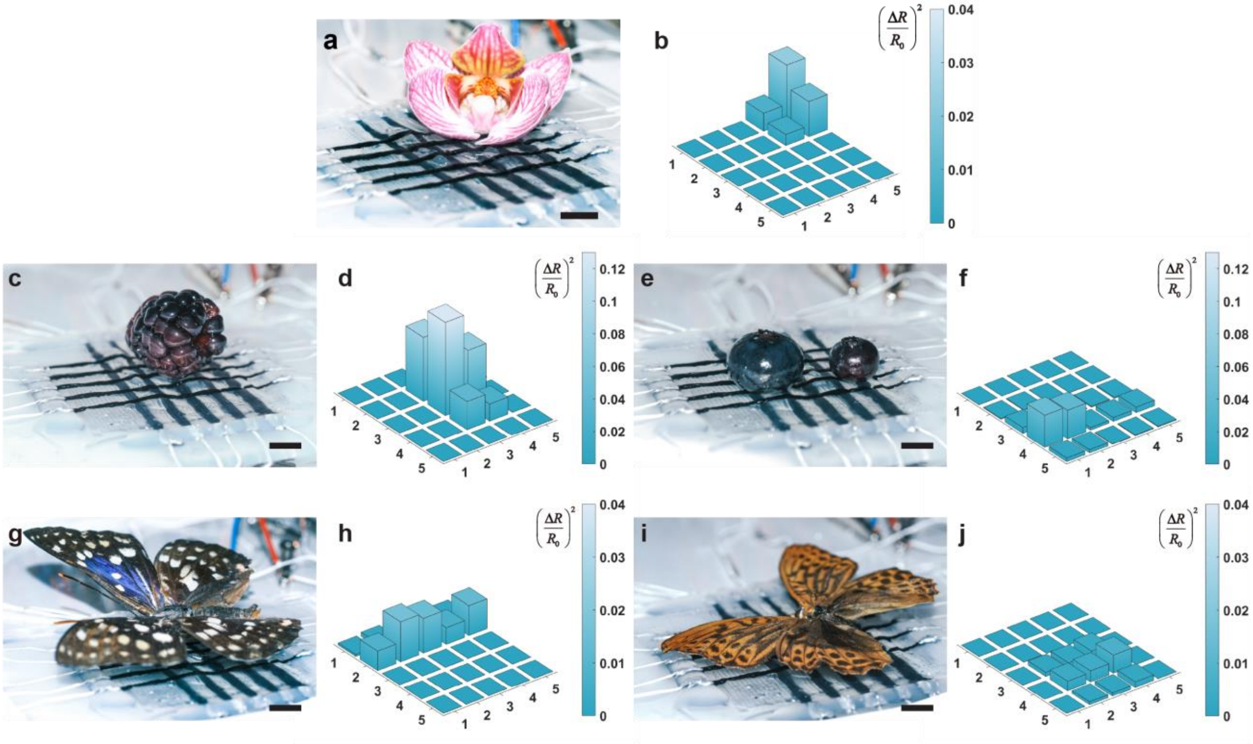


**Supplementary Fig. 31.** The sensory response for placing different objects on the sensor array. **a-b** An orchid (0.96g). **c-d** A blackberry (4.8 g). **e-f** Two blueberries (1.72 g and 0.58 g). **g-j** Butterfly specimens (0.55 g and 0.66 g). The scale bar is 5 mm.

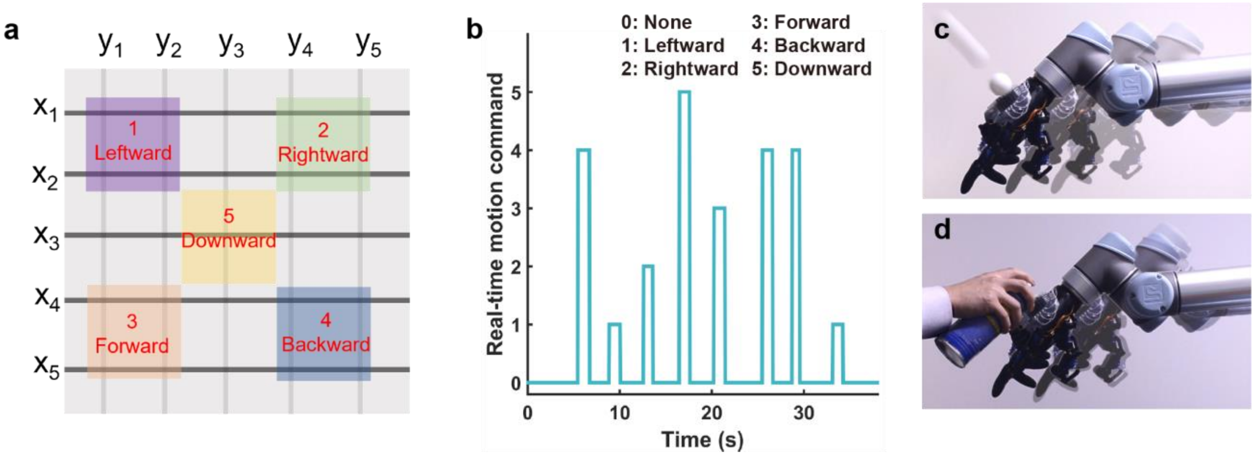


**Supplementary Fig. 32. a** The five parts separated on the sensor array to locate forces and generated commands. Specifically, when pressing part 1, the robot arm would move left ward. When part 2 is pressed, the robot arm would move rightward. Similarly, when pressing part 3, 4, 5, the robot arm would move forward, backward, and downward, respectively. **b** The real-time motion command sent by the sensory response of the robot e-skin. **c-d** Motions of the robot arm in response to the mechanical stimuli by a ping-pong ball or air spray.

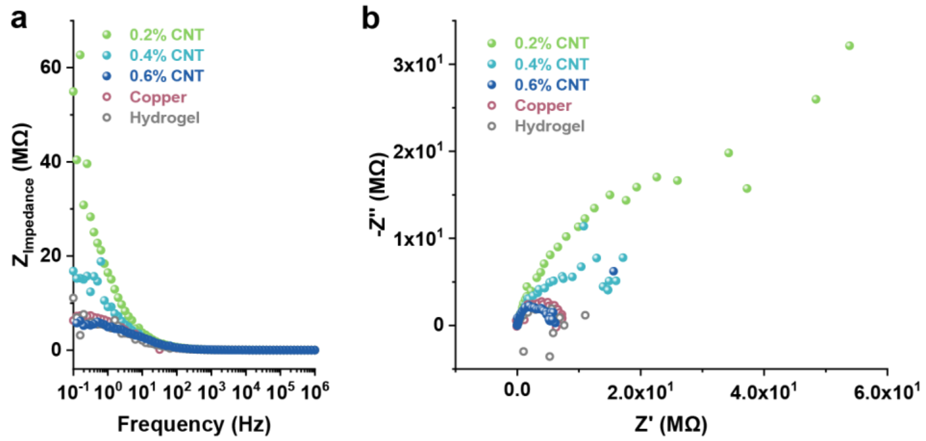


**Supplementary Fig. 33. a** Bode plot and **b** Nyquist plot (under the linear impedance axis) of skin impedance measurements using SWCNT/BBE, copper sheet, and hydrogels as electrodes on the skin.

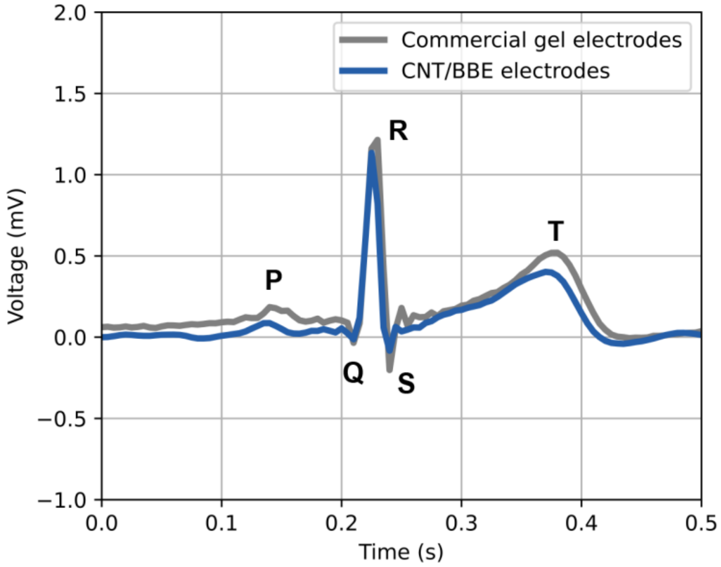


**Supplementary Fig. 34.** The overlayed ECG signal patterns measured by commercial gel electrodes and SWCNT/BBE electrodes.

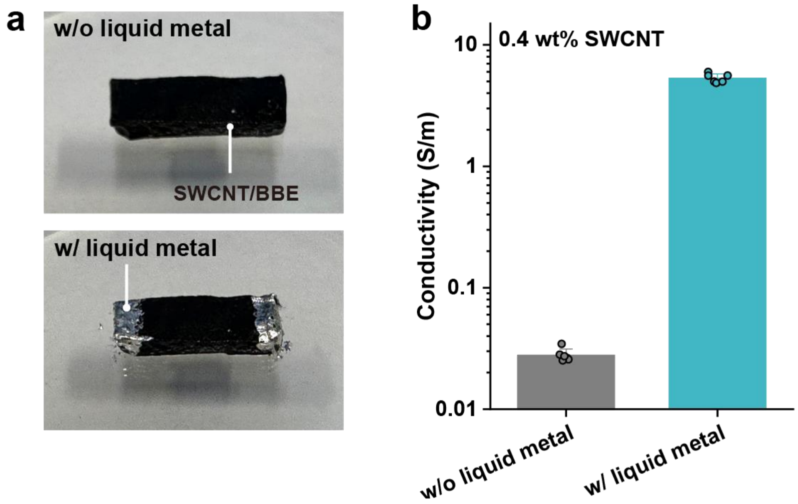


**Supplementary Fig. 35. a** Photographs of the 0.4 wt% SWCNT/BBE without and with liquid metal as the interface between the probe and samples. **b** Measured conductivity of 0.4 wt% SWCNT/BBE with and without using the liquid metal. Error bars denote the standard deviation of the measurements.

## Supplementary References


1. Vatankhah-Varnosfaderani, M. *et al.* Mimicking biological stress–strain behaviour with synthetic elastomers. *Nature* **549**, 497–501 (2017).
2. Duncan, T. T., Chan, E. P. & Beers, K. L. Maximizing Contact of Supersoft Bottlebrush Networks with Rough Surfaces to Promote Particulate Removal. *ACS Appl Mater Interfaces* **11**, 45310–45318 (2019).
3. Ina, M. *et al.* From adhesion to wetting: Contact mechanics at the surfaces of super-soft brush-like elastomers. *ACS Macro Lett* **6**, 854–858 (2017).
4. Jacobs, M. *et al.* Nonlinear Elasticity and Swelling of Comb and Bottlebrush Networks. *Macromolecules* **52**, 5095–5101 (2019).
5. Cai, L. H. *et al.* Soft Poly(dimethylsiloxane) Elastomers from Architecture-Driven Entanglement Free Design. *Advanced Materials* **27**, 5132–5140 (2015).
6. Cai, L. H. Molecular understanding for large deformations of soft bottlebrush polymer networks. *Soft Matter* **16**, 6259-6264 (2020).
7. Hu, P., Madsen, J. & Skov, A. L. One reaction to make highly stretchable or extremely soft silicone elastomers from easily available materials. *Nat. Commun.* **13**, 370 (2022).
8. Vatankhah-Varnosfaderani, M. *et al.* Chameleon-like elastomers with molecularly encoded strain-adaptive stiffening and coloration. *Science* **359**, 1509-1513 (2018).
9. Keith, A. N. *et al.* Bottlebrush Bridge between Soft Gels and Firm Tissues. *ACS Cent Sci* **6**, 413–419 (2020).
10. Keith, A. N. *et al.* Independently Tuning Elastomer Softness and Firmness by Incorporating Side Chain Mixtures into Bottlebrush Network Strands. *Macromolecules* **53**, 9306–9312 (2020).
11. Dashtimoghadam, E. *et al.* Injectable non-leaching tissue-mimetic bottlebrush elastomers as an advanced platform for reconstructive surgery. *Nat Commun* **12**, 1–11 (2021).

12. Reynolds, V. G. *et al.* Super-soft solvent-free bottlebrush elastomers for touch sensing. *Mater Horiz* **7**, 181–187 (2020).

13. Self, J. L. *et al.* Dynamic Bottlebrush Polymer Networks: Self-Healing in Super-Soft Materials. *J Am Chem Soc* **142**, 7567–7573 (2020).

14. Self, J. L. *et al.* Carbon Nanotube Composites with Bottlebrush Elastomers for Compliant Electrodes. *ACS Polymers Au* **2**, 27-34 (2021).

15. Xie, R. *et al.* Room temperature 3D printing of super-soft and solvent-free elastomers. *Sci Adv* **6**, 1–11 (2020).

16. Lee, K. *et al.* Stress-strain behavior of the electrospun thermoplastic polyurethane elastomer fiber mats. *Macromol Res* **13**, 441–445 (2005).

17. Lorenz, H. *et al.* Advanced elastomer nano-composites based on CNT-hybrid filler systems. *Compos Sci Technol* **69**, 2135–2143 (2009).

18. Zhang, L. *et al.* A Highly Efficient Self-Healing Elastomer with Unprecedented Mechanical Properties. *Advanced Materials* **31**, 1901402 (2019).

19. Seyedin, M. Z., Razal, J. M., Innis, P. C. & Wallace, G. G. Strain-responsive polyurethane/PEDOT:PSS elastomeric composite fibers with high electrical conductivity. *Adv Funct Mater* **24**, 2957–2966 (2014).

20. Buckley, C. P., Prisacariu, C. & Martin, C. Elasticity and inelasticity of thermoplastic polyurethane elastomers: Sensitivity to chemical and physical structure. *Polymer (Guildf)* **51**, 3213–3224 (2010).

21. Shi, C. Y. *et al.* An Ultrastrong and Highly Stretchable Polyurethane Elastomer Enabled by a Zipper-Like Ring-Sliding Effect. *Advanced Materials* **32**, 1–7 (2020).

22. Liao, Z., Hossain, M. & Yao, X. Ecoflex polymer of different Shore hardnesses: Experimental investigations and constitutive modelling. *Mechanics of Materials* **144**, (2020).

23. Bernardi, L., Hopf, R., Ferrari, A., Ehret, A. E. & Mazza, E. On the large strain deformation behavior of silicone-based elastomers for biomedical applications. *Polym Test* **58**, 189–198 (2017).

24. Tugui, C., Stiubianu, G. T., Serbulea, M. S. & Cazacu, M. Silicone dielectric elastomers optimized by crosslinking pattern-a simple approach to high-performance actuators. *Polym Chem* **11**, 3271–3284 (2020).

25. Kang, J. *et al.* Tough and Water-Insensitive Self-Healing Elastomer for Robust Electronic Skin. *Advanced Materials* **30**, 1–8 (2018).

26. Lee, W. S., Yeo, K. S., Andriyana, A., Shee, Y. G. & Mahamd Adikan, F. R. Effect of cyclic compression and curing agent concentration on the stabilization of mechanical properties of PDMS elastomer. *Mater Des* **96**, 470–475 (2016).

27. Zhang, B. *et al.* A Transparent, Highly Stretchable, Autonomous Self-Healing Poly(dimethyl siloxane) Elastomer. *Macromol Rapid Commun* **38**, (2017).

28. Liu, J. *et al.* Bioinspired engineering of two different types of sacrificial bonds into chemically cross-linked cis-1,4-polyisoprene toward a high-performance elastomer. *Macromolecules* **49**, 8593–8604 (2016).

29. Sedlačík, M. *Advances in elastomers*. *Materials* vol. 14 (2021).

30. Sahu, R. K., Patra, K., Bhaumik, S., Pandey, A. K. & Setua, D. K. Stress-Strain Behaviour of Dielectric Elastomer for Actuators. *Applied Mechanics and Materials* **789–790**, 837–841 (2015).

31. Merkel, D. R., Shaha, R. K., Yakacki, C. M. & Frick, C. P. Mechanical energy dissipation in polydomain nematic liquid crystal elastomers in response to oscillating loading. *Polymer (Guildf)* **166**, 148–154 (2019).

32. Song, X., Cao, L., Tanaka, R., Shiono, T. & Cai, Z. Optically Transparent Functional Polyolefin Elastomer with Excellent Mechanical and Thermal Properties. *ACS Macro Lett* **8**, 299–303 (2019).

33. Xie, R. *et al.* Room temperature 3D printing of super-soft and solvent-free elastomers. *Sci Adv* **6**, 1–11 (2020).

34. Choi, S. *et al.* Highly conductive, stretchable and biocompatible Ag–Au core–sheath nanowire composite for wearable and implantable bioelectronics. *Nat Nanotechnol* **13**, 1048–1056 (2018).

35. Fan, Y. J. *et al.* Highly conductive, stretchable, and breathable epidermal electrode based on hierarchically interactive nano-network. *Nanoscale* **12**, 16053–16062 (2020).

36. Li, Z. *et al.* Rational design of a printable, highly conductive silicone-based electrically conductive adhesive for stretchable radio-frequency antennas. *Adv Funct Mater* **25**, 464–470 (2015).

37. Ma, R., Kang, B., Cho, S., Choi, M. & Baik, S. Extraordinarily High Conductivity of Stretchable Fibers of Polyurethane and Silver Nanoflowers. *ACS Nano* **9**, 10876–10886 (2015).

38. Hyun, D. C. *et al.* Ordered zigzag stripes of polymer gel/metal nanoparticle composites for highly stretchable conductive electrodes. *Advanced Materials* **23**, 2946–2950 (2011).

39. Park, M. *et al.* Highly stretchable electric circuits from a composite material of silver nanoparticles and elastomeric fibres. *Nat Nanotechnol* **7**, 803–809 (2012).

40. Larmagnac, A., Eggenberger, S., Janossy, H. & Vörös, J. Stretchable electronics based on Ag-PDMS composites. *Sci Rep* **4**, 1–7 (2014).

41. Vural, M., Behrens, A. M., Ayyub, O. B., Ayoub, J. J. & Kofinas, P. Sprayable elastic conductors based on block copolymer silver nanoparticle composites. *ACS Nano* **9**, 336–344 (2015).

42. Park, J. *et al.* Electromechanical cardioplasty using a wrapped elasto-conductive epicardial mesh. *Sci Transl Med* **8**, 1–12 (2016).

43. Akter, T. & Kim, W. S. Reversibly stretchable transparent conductive coatings of spray-deposited silver nanowires. *ACS Appl Mater Interfaces* **4**, 1855–1859 (2012).

44. Lu, Y. *et al.* High-Performance Stretchable Conductive Composite Fibers from Surface-Modified Silver Nanowires and Thermoplastic Polyurethane by Wet Spinning. *ACS Appl Mater Interfaces* **10**, 2093–2104 (2018).

45. Tybrandt, K. *et al.* High-Density Stretchable Electrode Grids for Chronic Neural Recording. *Advanced Materials* **30**, (2018).

46. Kim, Y. *et al.* Stretchable nanoparticle conductors with self-organized conductive pathways. *Nature* **500**, 59–63 (2013).

47. Catenacci, M. J., Reyes, C., Cruz, M. A. & Wiley, B. J. Stretchable Conductive Composites from Cu-Ag Nanowire Felt. *ACS Nano* **12**, 3689–3698 (2018).

48. Bae, S., Ajmal, C. M., Lee, Y. & Baik, S. Significantly Enhanced Mechanical Strength by the Hollow Structure of Conductive Stretchable Silver Nanoflower-Polyurethane Fibers. *Adv Eng Mater* **22**, 1–9 (2020).

49. Kwon, C. *et al.* Self-Bondable and Stretchable Conductive Composite Fibers with Spatially Controlled Percolated Ag Nanoparticle Networks: Novel Integration Strategy for Wearable Electronics. *Adv Funct Mater* **30**, 1–10 (2020).

50. Cai, L. *et al.* Highly transparent and conductive stretchable conductors based on hierarchical reticulate single-walled carbon nanotube architecture. *Adv Funct Mater* **22**, 5238–5244 (2012).

51. Bhagavatheswaran, E. S. *et al.* Construction of an Interconnected Nanostructured Carbon Black Network: Development of Highly Stretchable and Robust Elastomeric Conductors. *Journal of Physical Chemistry C* **119**, 21723–21731 (2015).

52. Sekitani, T. *et al.* A rubberlike stretchable active matrix using elastic conductors. *Science* **321**, 1468-1472 (2008).

53. Sekitani, T. *et al.* Stretchable active-matrix organic light-emitting diode display using printable elastic conductors. *Nat Mater* **8**, 494–499 (2009).

54. Ma, T. *et al.* A Bioinspired Interface Design for Improving the Strength and Electrical Conductivity of Graphene-Based Fibers. *Advanced Materials* **30**, 1–8 (2018).

55. Fritzsche, J., Lorenz, H. & Klüppel, M. CNT Based Elastomer-Hybrid-Nanocomposites with Promising Mechanical and Electrical Properties. *Macromol Mater Eng* **294**, 551–560 (2009).

56. Wang, L. *et al.* Highly stretchable, anti-corrosive and wearable strain sensors based on the PDMS/CNTs decorated elastomer nanofiber composite. *Chemical Engineering Journal* **362**, 89–98 (2019).

57. Das, A. *et al.* Coupling activity of ionic liquids between diene elastomers and multi-walled carbon nanotubes. *Carbon N Y* **47**, 3313–3321 (2009).

58. Shin, M. K. *et al.* Elastomeric Conductive Composites Based on Carbon Nanotube Forests. *Advanced Materials* **22**, 2663–2667 (2010).

59. Kong, J. *et al.* Electrically conductive PDMS-grafted CNTs-reinforced silicone elastomer. *Compos Sci Technol* **159**, 208–215 (2018).

60. Zhang, C. *et al.* Generic Method to Create Segregated Structures toward Robust, Flexible, Highly Conductive Elastomer Composites. *ACS Appl Mater Interfaces* **13**, 24154–24163 (2021).

61. Hansen, T. S., West, K., Hassager, O. & Larsen, N. B. Highly stretchable and conductive polymer material made from poly(3,4-ethylenedioxythiophene) and polyurethane elastomers. *Adv Funct Mater* **17**, 3069–3073 (2007).

62. Lee, J. H. *et al.* Highly Conductive, Stretchable, and Transparent PEDOT:PSS Electrodes Fabricated with Triblock Copolymer Additives and Acid Treatment. *ACS Appl Mater Interfaces* **10**, 28027–28035 (2018).

63. Guan, Y. S., Zhang, Z., Tang, Y., Yin, J. & Ren, S. Kirigami-Inspired Nanoconfined Polymer Conducting Nanosheets with 2000% Stretchability. *Advanced Materials* **30**, (2018).

64. Lipomi, D. J. *et al.* Electronic properties of transparent conductive films of PEDOT:PSS on stretchable substrates. *Chemistry of Materials* **24**, 373–382 (2012).

65. Guo, L., Ma, M., Zhang, N., Langer, R. & Anderson, D. G. Stretchable polymeric multielectrode array for conformal neural interfacing. *Advanced Materials* **26**, 1427–1433 (2014).

66. Stoyanov, H., Kollosche, M., Risse, S., Waché, R. & Kofod, G. Soft conductive elastomer materials for stretchable electronics and voltage controlled artificial muscles. *Advanced Materials* **25**, 578–583 (2013).

67. Wang, Y. *et al.* A highly stretchable, transparent, and conductive polymer. *Sci Adv* **3**, 1–11 (2017).

68. Dauzon, E. *et al.* Stretchable and Transparent Conductive PEDOT:PSS-Based Electrodes for Organic Photovoltaics and Strain Sensors Applications. *Adv Funct Mater* **30**, 1–11 (2020).

69. Jia, L. C. *et al.* Stretchable Liquid Metal-Based Conductive Textile for Electromagnetic Interference Shielding. *ACS Appl Mater Interfaces* **12**, 53230–53238 (2020).

70. Mou, L. *et al.* Highly Stretchable and Biocompatible Liquid Metal-Elastomer Conductors for Self-Healing Electronics. *Small* **16**, 1–9 (2020).

71. Ma, Z. *et al.* Permeable superelastic liquid-metal fibre mat enables biocompatible and monolithic stretchable electronics. *Nat Mater* **20**, 859–868 (2021).

72. Veerapandian, S. *et al.* Hydrogen-doped viscoplastic liquid metal microparticles for stretchable printed metal lines. *Nat Mater* **20**, 533–540 (2021).

73. Wang, J. *et al.* Printable Superelastic Conductors with Extreme Stretchability and Robust Cycling Endurance Enabled by Liquid-Metal Particles. *Advanced Materials* **30**, 24–26 (2018).

74. Silva, A. F. *et al.* High Resolution Soft and Stretchable Circuits with PVA/Liquid-Metal Mediated Printing. *Adv Mater Technol* **5**, 1–9 (2020).

75. Markvicka, E. J., Bartlett, M. D., Huang, X. & Majidi, C. An autonomously electrically self-healing liquid metal-elastomer composite for robust soft-matter robotics and electronics. *Nat Mater* **17**, 618–624 (2018).

76. Liu, S. *et al.* Laser Sintering of Liquid Metal Nanoparticles for Scalable Manufacturing of Soft and Flexible Electronics. *ACS Appl Mater Interfaces* **10**, 28232–28241 (2018).

77. Luo, J. Q. *et al.* Flexible, stretchable and electrically conductive MXene/natural rubber nanocomposite films for efficient electromagnetic interference shielding. *Compos Sci Technol* **182**, 107754 (2019).

78. Aakyiir, M. *et al.* Electrically and thermally conductive elastomer by using MXene nanosheets with interface modification. *Chemical Engineering Journal* **397**, 125439 (2020).

79. Self, J. L. *et al.* Carbon Nanotube Composites with Bottlebrush Elastomers for Compliant Electrodes. *ACS Polymers Au* **2**, 27–34 (2022).
