# Supplementary material for: Conductive and elastic bottlebrush elastomers for ultrasoft electronics
Source: Nat Commun. 2023 Feb 4;14:623. doi: 10.1038/s41467-023-36214-8 (PMC9899285; doi:10.1038/s41467-023-36214-8)
Supplement: Supplementary file 3 — Description of Additional Supplementary Files [file 41467_2023_36214_MOESM3_ESM.docx]

**Description of Additional Supplementary Files for**

Conductive and elastic bottlebrush elastomers for ultrasoft electronics

Pengfei Xu, Shaojia Wang**,** Angela Lin, Hyun-Kee Min, Zhanfeng Zhou, Wenkun Dou, Yu Sun, Xi Huang, Helen Tran*, Xinyu Liu*

*Corresponding authors. Email: [tran@utoronto.ca](mailto:tran@utoronto.ca); [xyliu@mie.utoronto.ca](mailto:xyliu@mie.utoronto.ca)

File name: Supplementary Movie 1

Description: Adhesive property of PDMS BBE (MM:CL = 1200:1)

File name: Supplementary Movie 2

Description: Adhesive property of SWCNT/BBE

File name: Supplementary Movie 3

Description: Bonding between the SWCNT/BBE and pure BBE

File name: Supplementary Movie 4

Description: The low impact of the SWCNT/BBE strain sensor on the actuation of soft actuator

File name: Supplementary Movie 5

Description: The attachment of the laser-cut strain sensor on the human body

File name: Supplementary Movie 6

Description: The physical effect of attaching the SWCNT/BBE and PDMS Sylgard 184 on the hornworm

File name: Supplementary Movie 7

Description: Applying SWCNT/BBE as wearable electronics for the hornworm

File name: Supplementary Movie 8

Description: The sensory response of the 3D-printed touch pad by placing a flower petal

File name: Supplementary Movie 9

Description: Human-Machine Interaction: 3D-printed touch pad as the “robotic e-skin”
